# Risk of Ventricular Arrhythmia with Citalopram and Escitalopram: A Population-Based Study

**DOI:** 10.1371/journal.pone.0160768

**Published:** 2016-08-11

**Authors:** Elena Qirjazi, Eric McArthur, Danielle M. Nash, Stephanie N. Dixon, Matthew A. Weir, Akshya Vasudev, Racquel Jandoc, Lorne J. Gula, Matthew J. Oliver, Ron Wald, Amit X. Garg

**Affiliations:** 1 Division of Nephrology, Department of Medicine, Western University, London, Ontario, Canada; 2 Institute for Clinical Evaluative Sciences, Toronto, Ontario, Canada; 3 Department of Epidemiology & Biostatistics, Western University, London, Ontario, Canada; 4 Division of Geriatric Psychiatry, Department of Psychiatry, Western University, London, Ontario, Canada; 5 Division of Clinical Pharmacology, Department of Medicine, Western University, London, Ontario, Canada; 6 Division of Cardiology, Department of Medicine, Western University, London, Ontario, Canada; 7 Department of Medicine, Sunnybrook Health Sciences Centre, University of Toronto, Toronto, Ontario, Canada; 8 Keenan Research Centre in the Li Ka Shing Knowledge Institute of St Michael’s Hospital, Toronto, Ontario, Canada; 9 Division of Nephrology, Department of Medicine, University of Toronto, Toronto, Ontario, Canada; Texas Technical University Health Sciences Center, UNITED STATES

## Abstract

**Background:**

The risk of ventricular arrhythmia with citalopram and escitalopram is controversial. In this study we investigated the association between these two drugs and the risk of ventricular arrhythmia.

**Methods:**

We conducted a population-based retrospective cohort study of older adults (mean age 76 years) from 2002 to 2012 in Ontario, Canada, newly prescribed citalopram (n = 137 701) or escitalopram (n = 38 436), compared to those prescribed referent antidepressants sertraline or paroxetine (n = 96 620). After inverse probability of treatment weighting using a propensity score, the baseline characteristics of the comparison groups were similar. The primary outcome was a hospital encounter with ventricular arrhythmia within 90 days of a new prescription, assessed using hospital diagnostic codes. The secondary outcome was all-cause mortality within 90 days.

**Results:**

Citalopram was associated with a higher risk of a hospital encounter with ventricular arrhythmia compared with referent antidepressants (0.06% vs. 0.04%, relative risk [RR] 1.53, 95% confidence intervals [CI]1.03 to 2.29), and a higher risk of mortality (3.49% vs. 3.12%, RR 1.12, 95% CI 1.06 to 1.18). Escitalopram was not associated with a higher risk of ventricular arrhythmia compared with the referent antidepressants (0.03% vs. 0.04%, RR 0.84, 95% CI 0.42 to 1.68), but was associated with a higher risk of mortality (2.86% vs. 2.63%, RR 1.09, 95% CI 1.01 to 1.18).

**Conclusion:**

Among older adults, initiation of citalopram compared to two referent antidepressants was associated with a small but statistically significant increase in the 90-day risk of a hospital encounter for ventricular arrhythmia.

## Introduction

Selective serotonin re-uptake inhibitors (SSRIs; e.g., citalopram, escitalopram, paroxetine and sertraline) are commonly prescribed antidepressants.[1–4] Citalopram and escitalopram have been implicated in ventricular arrhythmias, presumably by lengthening the QT interval of the cardiac cycle.[5–18] The Food and Drug Administration (FDA) and Health Canada caution against the use of citalopram at doses >20 mg/day in patients over 65 years of age).[19–22] The FDA warnings were based on an unpublished trial of 119 patients randomized to placebo or citalopram, demonstrating an increase in the corrected QT interval with citalopram.[19] These safety warnings have been controversial,[23–25] with inconsistent findings in other follow-up studies.[5, 14, 23, 26–29] Many of these studies were limited by the use of QT prolongation rather than ventricular arrhythmia risk,[14, 26, 28] a young population cohort,[5, 23, 27, 28] low statistical power,[26] and not accounting for important confounding factors in the analysis.[29] Escitalopram (the S enantiomer of citalopram) has also been associated with QT interval prolongation and Health Canada warns against the use of >10mg/day of escitalopram for patients 65 years of age or older.[5, 7, 14–15, 28, 30] We conducted this large propensity score-weighted population-based cohort study of older adults to investigate whether initiating citalopram or escitalopram in the outpatient setting is associated with a higher risk of ventricular arrhythmia, compared to initiating sertraline or paroxetine (referent antidepressants).

## Methods

### Design and Setting

We conducted a population-based retrospective cohort study of older adults from April 1, 2002 to December 31, 2012 in Ontario, Canada, who had received a new outpatient prescription for citalopram, escitalopram, sertraline or paroxetine (the most commonly prescribed SSRIs in Ontario). Ontario has approximately 2 million residents 65 years of age or older, who have full coverage for hospital and physician services, and prescription drugs.[31]

We used datasets held securely in linkable-files without any direct personal identifiers, and analyzed at the Institute for Clinical Evaluative Sciences (ICES). Patient information was anonymized and de-identified prior to analysis. The pre-specified protocol was approved by the Research Ethics Board at Sunnybrook Health Sciences Centre (Toronto, Ontario, Canada). The reporting of this study follows guidelines for observational studies (see S1 Table).[32]

### Data Sources

We ascertained patient baseline characteristics, drug use and outcome data using records from eight linked databases. The Ontario Drug Benefit database contains highly accurate records for outpatient prescriptions dispensed to patients aged 65 years or older (error rate less than 1%).[33] The Ontario Registered Persons Database records vital statistics, including date of death. The Canadian Institute for Health Information (CIHI)–Discharge Abstract Database, the CIHI—National Ambulatory Care Reporting System database, and the Ontario Mental Health Reporting System database contain diagnostic and procedural information on all hospitalizations, emergency room and psychiatric facility visits. The ICES Physician Database reports prescriber and specialist referral data. The Ontario Health Insurance Plan database (OHIP) includes health claims for physician services, and the Canadian Organ Replacement Register identifies patients with end-stage kidney disease. We have used these databases previously to research adverse drug events and health outcomes.[34–40] The information obtained was complete, except for neighbourhood income quintile (missing in 0.3% of patients) and prescriber specialty (missing in 12.7% of patients).

We used *International Classification of Diseases 9*^*th*^ *revision* (ICD 9; pre-2002) and *10*^*th*^ *revision* (ICD 10; post- 2002) codes to assess baseline co-morbidities in the five years prior to the receipt of the relevant prescriptions (S2 Table), in concordance with prior studies [34, 36]. We assessed baseline medications and health care use in the 120 days and 1 year prior to the date of the new SSRI prescription, respectively.

### Patients

We established a cohort of older adults in Ontario, Canada, who were dispensed a new outpatient prescription of at least 7 days for citalopram, escitalopram, paroxetine or sertraline between April 2002 and December 2012. The prescription date was the cohort entry date. Patients were separated into three groups based on their prescription: 1) citalopram, 2) escitalopram and 3) referent antidepressants (paroxetine or sertraline). Paroxetine and sertraline were grouped together as they have low cardiac toxicity, and both are prescribed for similar indications as citalopram and escitalopram.[10, 14–15, 27]

We excluded from analyses: patients in their first year of eligibility for prescription drug coverage (age 65) to avoid incomplete medication records; those with antidepressant prescriptions in the 180 days prior to the cohort entry date to ensure new antidepressant use; those discharged from hospital in the two days prior to their cohort entry date to ensure new outpatient prescriptions (patients continuing antidepressants initiated in hospital would have their outpatient prescription dispensed on the same day or the day after hospital discharge); those with a history of ventricular arrhythmia, cardiac arrest or implantable cardiac defibrillator to capture *de novo* arrhythmic events in follow up; and those with nonstandard daily drug doses to ensure generalizability to usual care and omit data errors. Patients with multiple eligible study drug prescriptions entered the cohort once on the first prescription.

### Outcomes

We ascertained all outcomes within 90 days of the cohort entry date, to mimic the duration of follow-up in corresponding clinical trials and to avoid potential crossovers between the groups that could occur with longer follow up.[5, 14] Since, QT prolongation starts within hours of initiating citalopram or escitalopram, we expected drug-related ventricular arrhythmias to occur soon after SSRI initiation.[7, 41–42]

The primary outcome was at least one hospital encounter (emergency room presentation or hospital admission) with ventricular arrhythmia. The secondary outcome was all-cause mortality. Diagnostic codes used to ascertain outcomes are listed in S3 Table (*ICD 10* diagnostic codes were used to assess ventricular arrhythmia and this coding system was implemented in Canada in 2002). These codes are entered into the databases by trained personnel based on physician-recorded diagnoses in patients’ medical charts. The *ICD 10* codes for ventricular arrhythmia have not been previously validated. However, their sensitivity is expected to be low as ventricular arrhythmias frequently go undetected in routine healthcare (often occuring outside hospital settings, in unmonitored patients, or in the setting of multi-organ medical illness). Previous studies assessing the accuracy of ICD 9 and ICD 10 codes for cardiac arrhythmia (ventricular and supraventricular) show a positive predictive value exceeding 80%.[43–46] We performed an ethics-approved manual review of 202 random charts in our region, looking at hospital encounters (emergency visits or admissions) with the ventricular arrhythmia codes used in this study, and confirmed a positive predictive value of 92% (95% confidence interval [CI] 87 to 95%). All-cause mortality data is accurately coded in our data sources, with a sensitivity of 97.8% and specificity of 100% for the finding of death.[47]

### Statistical Analysis

We used inverse probability of treatment weights based on propensity scores to eliminate systematic differences in the baseline characteristics of the compared groups while retaining all individuals in the analysis.[48] The propensity scores provided the probability of receiving a prescription for the exposure drug (citalopram or escitalopram) given a set of measured baseline characteristics. Scores were calculated using multivariable logistic regression models with 48 baseline characteristics (S4 Table)–chosen because of their potential influence on the outcomes or segregation of patients between the compared groups.[49–51] By applying propensity score-based weights to the patients and outcomes, we created weighted groups that were well-balanced on all measured baseline characteristics. We compared baseline characteristics between the groups using standardized differences. This metric describes differences between the group means relative to the pooled standard deviation—a difference greater than 10% is considered meaningful.[48, 52] We calculated relative risks (RR) with 95% confidence intervals (CI) using log-binomial regression models accounting for the weights.

We also evaluated the association for both exposed groups (citalopram and escitalopram) with our outcomes in pre-specified subgroups of patients—defined by the presence or absence of: 1) congestive heart failure, 2) coronary artery disease, 3) chronic kidney disease, and 4) high dose (S5 Table). We hypothesized a higher risk in the presence of these conditions. We identified chronic kidney disease using an algorithm of hospital diagnostic codes validated for older adults in our region.[53] We determined interaction p-values by including interaction terms in the regression models. We interpreted a two-sided p-value of less than 0.05 as statistically significant, and performed all analysis using SAS version 9.3 (SAS Institute, Cary, North Carolina).

## Results

### Baseline Characteristics

We identified 472 001 patients with prescriptions for the study SSRIs. After applying our selection criteria, we had 137 701 older adults with prescriptions for citalopram, 38 436 for escitalopram and 96 620 for the referent antidepressants (refer to S1 Fig; less than 10% of patients [40 015 patients] were excluded for non-standard daily doses of the SSRI). The mean age was 76 years old (range 66 to 105), and 66% were women. General practitioners wrote 78% of the prescriptions. The distribution of baseline characteristics before and after propensity score weighting are presented in Table 1 for citalopram and Table 2 for escitalopram.

**Table 1:** Baseline characteristics for the citalopram cohort (pre and post weighting).

	Un-weighted			Weighted^a^		
	Citalopram n = 137 701 (%)	Paroxetine or Sertraline n = 96 620 (%)	Standardized Difference^b^ (%)	Citalopram n = 137 701 (%)	Paroxetine or Sertralinen = 135 746(%)	Standardized Difference^b^ (%)
**DEMOGRAPHICS**						
Age, years	76 (7.4)	75 (7.1)	19	76 (7.4)	76 (8.7)	2
Women	65.5	66.8	3	65.5	65.8	1
Rural^**c**^	15.4	13.5	5	15.4	15.3	0
Long term care	6.9	3.4	16	6.9	6.3	2
Income quintile^d^						
One (lowest)	20.6	20.9	1	20.6	20.8	0
Two	21.2	21.9	2	21.2	21.8	1
Three (medium)	19.7	20.0	1	19.7	20.1	1
Four	19.0	18.6	1	19.0	18.5	1
Five (highest)	19.5	18.6	2	19.5	18.7	2
Year of cohort entry^e^						
2002–2005	41.6	60.7	39	41.6	58.1	33
2006–2009	39.8	25.1	32	39.8	26.7	28
2010–2012	18.6	14.2	12	18.6	15.2	9
**COMORBIDITIES**^f^						
Charlson Comorbidity Index^g^	0.74 (1.1)	0.62 (1.0)	12	0.74 (1.1)	0.73 (1.3)	1
Dementia	20.3	12.5	21	20.3	19.2	2
Schizophrenia/psychotic disorders	4.0	3.4	3	4.0	4.4	2
Bipolar disorder	3.6	3.5	0	3.6	3.8	1
Unipolar depression/anxiety disorder^h^	21.6	20.6	2	21.6	21.0	1
History of self-harm	0.2	0.1	2	0.2	0.1	1
Major haemorrhage	5.7	4.7	4	5.7	5.6	0
Haemorrhagic Stroke	0.6	0.4	3	0.6	0.5	1
Ischemic Stroke	4.7	3.2	7	4.7	4.5	1
Transient Ischemic Attack	1.3	1.1	2	1.3	1.5	1
Chronic liver disease	3.7	3.6	1	3.7	3.8	0
Chronic Kidney Disease	6.7	5.1	7	6.7	6.5	1
Congestive heart failure	15.8	13.7	6	15.8	15.7	0
Coronary artery disease^i^	32.4	30.6	4	32.4	32.3	0
Angina	24.0	23.5	1	24.0	24.0	0
Acute myocardial infarction	4.5	3.9	3	4.5	4.5	0
Pacemaker	3.2	2.4	5	3.2	3.1	0
Atrial fibrillation/flutter	4.7	4.2	2	4.7	4.7	0
Peripheral vascular disease	2.3	2.1	1	2.3	2.5	1
Chronic lung disease	30.7	30.5	0	30.7	30.8	0
Cancer^j^	15.7	14.1	5	15.7	15.7	0
Alcoholism	2.4	2.4	0	2.4	2.5	0
Seizure	1.0	0.8	2	1.0	1.0	0
Acute kidney injury	2.4	1.6	6	2.4	2.3	1
Hospitalization with hyperkalemia	0.8	0.7	2	0.8	0.9	1
Venous Thromboembolism	1.5	1.1	3	1.5	1.5	0
**MEDICATIONS**^k^						
Anti-arrhythmics	2.2	2.1	1	2.2	2.3	1
Antipsychotics	7.1	5.0	9	7.1	6.9	1
Proton pump inhibitors	31.2	27.1	9	31.2	31.0	0
Anti-emetic	2.2	1.7	3	2.2	2.0	1
Lithium	0.3	0.3	1	0.3	0.3	0
Anti-lipemics	41.6	39.4	5	41.6	41.2	1
Antihypertensives	72.1	68.8	7	72.1	71.9	0
H2RAs	10.4	12.8	8	10.4	10.5	0
Pro-kinetics	4.6	4.1	3	4.6	4.5	1
Antidiabetics	16.3	15.0	3	16.3	16.0	1
Acetylsalicylic acid	9.6	11.5	6	9.6	9.7	0
Anticoagulants	10.4	7.7	9	10.4	10.2	1
Antiplatelet	6.5	4.7	8	6.5	6.3	1
Tri-cyclic antidepressants	1.4	1.6	1	1.4	1.5	1
Opioids	0.0	0.1	1	0.0	0.1	1
Anti-malarial	0.7	0.7	0	0.7	0.7	0
Anti-viral	0.0	0.0	0	0.0	0.0	1
Antibiotic	36.6	36.4	1	36.6	36.9	1
Antineoplastic	4.5	3.8	4	4.5	4.1	2
Benzodiazepine	39.6	42.8	5	39.6	40.3	1
NSAIDS^l^	21.5	24.0	6	21.5	21.7	1
Cholinesterase inhibitors	0.0	0.0	0	0.0	0.0	0
Anticonvulsants	3.6	3.0	4	3.6	3.4	1
**DOSE**^m^						
High	6.5	12.6	21	6.5	6.8	1
**PRESCRIBER**						
General Practitioner	76.6	78.5	5	76.6	76.9	1
Psychiatrist	2.6	2.4	1	2.6	2.6	0
Internist	0.8	0.7	1	0.8	0.8	0
Other	6.9	4.8	9	6.9	6.5	2
Missing	13.1	13.5	1	13.1	13.3	1
**HEALTH CARE USE**^n^						
Number of Hospitalizations						
0	58.9	63.9	10	58.9	59.8	2
1 to 3	37.2	33.0	9	37.2	36.3	2
4 to 6	3.4	2.6	5	3.4	3.3	1
7 to 9	0.4	0.3	2	0.4	0.4	0
10 to 12	0.1	0.1	0	0.1	0.1	0
over 12	0.0	0.0	0	0.0	0.1	1
Number of Emergency room visits						
0	52.7	59.5	14	52.7	54.3	3
1 to 3	39.7	34.6	11	39.7	37.9	4
Over 3	7.6	13.0	18	7.6	7.8	1
General Practitioner Visits						
0–4	13.9	15.9	6	13.9	14.5	2
5–9	22.4	24.2	4	22.4	22.2	0
10–14	19.5	20.2	2	19.5	19.2	1
15–19	12.9	13.0	0	12.9	12.9	0
20–24	8.6	8.2	1	8.6	8.5	0
25–29	5.7	5.3	2	5.7	5.7	0
≥30	17.1	13.2	11	17.1	16.9	1
At home physician services	11.0	10.2	3	11.0	11.7	2
Specialist Consultations						
Psychiatrist consults	7.8	6.0	7	7.8	7.6	1
Nephrologist consults^o^	6.0	5.1	4	6.0	6.0	0
Cardiologist visits	42.1	37.9	9	42.1	41.7	1
Neurologist consults	11.5	9.5	7	11.5	11.3	1
Diagnostic tests/Interventions						
Electrocardiogram	87.8	86.2	5	87.8	87.6	0
Stress test	35.3	34.9	1	35.3	35.0	0
Echocardiography	41.7	38.1	7	41.7	41.4	1
Cardiac Catheterization	6.8	6.3	2	6.8	6.5	1
Holter Monitor	21.1	19.3	5	21.1	21.0	0
Coronary angiogram	7.5	6.8	3	7.5	7.0	2
Chest X-ray	78.4	76.1	5	78.4	78.2	0
Pulmonary function test	24.8	25.0	0	24.8	25.1	1
Carotid ultrasound	19.5	17.2	6	19.5	19.3	1
Computed Tomography of the Head	37.0	29.7	15	37.0	36.1	2
Computed Tomography of other area	35.7	30.2	12	35.7	35.3	1
Mammogram	27.0	30.7	8	27.0	27.4	1
Bone Mineral Density	40.1	39.6	1	40.1	40.3	0

Data presented as percent except for age and Charlson Comorbidity Index which are presented as mean (standard deviation).

***Abbreviations***: Non-steroidal anti-inflammatory drug (NSAID)–excludes acetyl-salicylic acid, Acetylsalicylic acid (ASA), Histamine H2 Receptor antagonist (H2RA), Not applicable (N/A)

^a^ Weighted cohort based on inverse probability of treatment weights, using a propensity score based on 48 baseline characteristics.

^b^ Standardized differences are less sensitive to sample size than traditional hypothesis tests. They provide a measure of the difference between groups divided by the pooled standard deviation; a value greater than 10% is interpreted as a meaningful difference between the groups.

^c^ Defined as a population <10 000 people.

^d^ Income was categorized into fifths of average neighbourhood income on the cohort entry date.

^e^ The year of cohort entry is also referred to as the year of cohort entry date.

^f^ Comorbidities assessed by administrative database codes in the previous 5 years.

^g^ Charlson Comorbidity Index [Charlson ME, Pompei P, Alex KL, Mackenzie CR. A new method for classifying prognostic comorbidity in longitudinal studies: development and validation. J Chron Dis 1987;40(5):373–383. Quan H, Sundararajan V, Halfon P, Fong A, Burnand B, Luthi JC, et al. Coding algorithms for defining comorbidities in ICD-9-CM and ICD-10 administrative data. Med Care 2005;43(11):1130–1139.] was calculated using 5 years of hospitalization data. “No hospitalizations” received a score of 0.

^h^ The prevalence of depression is low since depression is not usually an in-patient disorder, and thus often not coded in the source databases.

^i^ Coronary artery disease includes receipt of coronary artery bypass graft surgery and percutaneous coronary intervention.

^j^ Major cancers include esophagus, lung, bowel, liver, pancreas, breast, male/female reproductive organs, as well as leukemias and lymphomas.

^k^ Baseline medication use assessed in the previous 120 days.

^l^ Excludes acetylsalicylic acid.

^m^ Refer to S5 Table for definitions of high and low doses.

^n^ Health care use assessed in the one year prior to SSRI prescription.

^o^ Based on the ICES physician database.

**Table 2:** Baseline characteristics for the escitalopram cohort (pre and post weighting).

	Un-weighted			Weighted^a^		
	Escitalopram n = 38 436 (%)	Paroxetine or Sertraline n = 96 620 (%)	Standardized Difference^b^ (%)	Escitalopram n = 38 436 (%)	Paroxetine or Sertraline n = 113 058 (%)	Standardized Difference^b^ (%)
**DEMOGRAPHICS**						
Age, years	76 (7.6)	75 (7.1)	9	76 (7.6)	75 (4.5)	4
Women	63.0	66.8	8	63.0	63.2	0
Rural^c^	12.8	13.5	2	12.8	12.8	0
Long term care	5.0	3.4	8	5.0	4.6	2
Income quintile^d^						
One (lowest)	18.9	20.9	5	18.9	20.0	3
Two	20.3	21.9	4	20.3	21.4	3
Three (medium)	19.6	20.0	1	19.6	20.2	2
Four	20.1	18.6	4	20.1	19.0	3
Five (highest)	21.1	18.6	6	21.1	19.3	4
Year of cohort entry^e^						
2002–2005	0.0	60.7	176	0.0	49.0	139
2006–2009	20.4	25.1	11	20.4	30.5	23
2010–2012	79.6	14.2	173	79.6	20.6	146
**COMORBIDITIES**^f^						
Charlson Comorbidity Index^g^	0.65 (1.1)	0.62 (1.0)	4	0.65 (1.1)	0.64 (0.7)	2
Dementia	19.8	12.5	21	19.8	18.6	4
Schizophrenia/psychotic disorders	3.9	3.4	2	3.9	4.3	3
Bipolar disorder	4.0	3.5	2	4.0	4.3	2
Unipolar depression/anxiety disorder^h^	19.8	20.6	2	19.8	20.8	3
History of self-harm	0.2	0.1	3	0.2	0.1	2
Major haemorrhage	6.3	4.7	7	6.3	6.1	1
Haemorrhagic Stroke	0.4	0.4	1	0.4	0.5	1
Ischemic Stroke	2.8	3.2	2	2.8	2.7	1
Transient Ischemic Attack	0.8	1.1	3	0.8	1.3	6
Chronic liver disease	3.7	3.6	0	3.7	4.0	2
Chronic Kidney Disease	7.2	5.1	9	7.2	6.9	1
Congestive heart failure	11.9	13.7	5	11.9	11.7	1
Coronary artery disease^i^	28.3	30.6	5	28.3	27.9	1
Angina	18.3	23.5	13	18.3	18.3	0
Acute myocardial infarction	3.6	3.9	1	3.6	3.6	0
Pacemaker	3.2	2.4	5	3.2	3.1	1
Atrial fibrillation/flutter	2.9	4.2	7	2.9	3.5	4
Peripheral vascular disease	1.6	2.1	4	1.6	2.1	5
Chronic lung disease	28.7	30.5	4	28.7	28.6	0
Cancer^j^	15.3	14.1	3	15.3	15.3	0
Alcoholism	2.3	2.4	0	2.3	2.4	0
Seizure	0.7	0.8	2	0.7	0.8	3
Acute kidney injury	2.8	1.6	9	2.8	2.6	1
Hospitalization with hyperkalemia	0.7	0.7	0	0.7	0.8	2
Venous Thromboembolism	1.3	1.1	1	1.3	1.3	1
**MEDICATIONS**^k^						
Anti-arrhythmics	1.5	2.1	5	1.5	2.1	5
Antipsychotics	7.1	5.0	9	7.1	6.9	1
Proton pump inhibitors	35.5	27.1	18	35.5	35.5	0
Anti-emetic	2.0	1.7	2	2.0	1.8	2
Lithium	0.3	0.3	0	0.3	0.4	2
Anti-lipemics	50.6	39.4	23	50.6	50.4	1
Antihypertensives	70.7	68.8	4	70.7	70.3	1
H2RAs	5.2	12.8	25	5.2	5.2	0
Pro-kinetics	4.5	4.1	2	4.5	4.5	0
Antidiabetics	17.7	15.0	7	17.7	17.5	1
Acetylsalicylic acid	4.4	11.5	24	4.4	4.4	0
Anticoagulants	9.4	7.7	6	9.4	9.2	1
Antiplatelet	7.5	4.7	12	7.5	7.4	0
Tri-cyclic antidepressants	1.1	1.6	4	1.1	1.5	4
Opioids	0.0	0.1	1	0.0	0.1	1
Anti-malarial	0.7	0.7	0	0.7	0.7	0
Anti-viral	0.0	0.0	0	0.0	0.0	1
Antibiotic	35.5	36.4	2	35.5	36.4	3
Antineoplastic	4.3	3.8	3	4.3	4.0	2
Benzodiazepine	35.5	42.8	13	35.5	36.0	1
NSAIDS^l^	17.4	24.0	16	17.4	17.5	0
Cholinesterase inhibitors	0.0	0.0	0	0.0	0.0	0
Anticonvulsants	4.0	3.0	6	4.0	3.4	5
**DOSE**^m^						
High	9.0	12.6	11	9.0	9.4	2
**PRESCRIBER**						
General Practitioner	81.0	78.5	6	81.0	81.2	1
Psychiatrist	4.5	2.4	12	4.5	4.5	0
Internist	0.5	0.7	3	0.5	0.5	0
Other	5.1	4.8	1	5.1	4.8	1
Missing	8.9	13.5	15	8.9	9.0	0
**HEALTH CARE USE**^n^						
Number of Hospitalizations						
0	63.1	63.9	2	63.1	62.9	0
1 to 3	33.8	33.0	2	33.8	34.2	1
4 to 6	2.7	2.6	1	2.7	2.5	1
7 to 9	0.3	0.3	0	0.3	0.3	0
10 to 12	0.1	0.1	0	0.1	0.0	0
over 12	0.0	0.0	0	0.0	0.0	0
Number of Emergency room visits						
0	54.5	59.5	10	54.5	56.1	3
1 to 3	38.4	34.6	8	38.4	36.9	3
Over 3	7.1	13.0	20	7.1	7.0	0
General Practitioner Visits						
0–4	14.6	15.9	4	14.6	14.6	0
5–9	25.5	24.2	3	25.5	23.8	4
10–14	21.5	20.2	3	21.5	20.2	3
15–19	12.7	13.0	1	12.7	13.2	1
20–24	7.8	8.2	1	7.8	8.5	3
25–29	4.7	5.3	3	4.7	5.5	4
≥30	13.2	13.2	0	13.2	14.2	3
At home physician services	7.8	10.2	8	7.8	9.9	10
Specialist Consultations						
Psychiatrist consults	9.1	6.0	12	9.1	8.9	1
Nephrologist consults^o^	7.1	5.1	8	7.1	7.1	0
Cardiologist visits	44.9	37.9	14	44.9	44.8	0
Neurologist consults	10.0	9.5	2	10.0	10.0	0
Diagnostic tests/Interventions						
Electrocardiogram	89.1	86.2	8	89.1	88.9	1
Stress test	39.1	34.9	9	39.1	37.4	5
Echocardiography	47.7	38.1	20	47.7	47.4	1
Cardiac Catheterization	7.1	6.3	3	7.1	5.9	7
Holter Monitor	24.2	19.3	12	24.2	24.0	1
Coronary angiogram	8.0	6.8	5	8.0	6.4	8
Chest X-ray	77.7	76.1	4	77.7	77.4	1
Pulmonary function test	26.7	25.0	4	26.7	26.6	0
Carotid ultrasound	20.0	17.2	7	20.0	19.7	1
Computed Tomography of the Head	37.8	29.7	17	37.8	37.1	2
Computed Tomography of other area	41.8	30.2	25	41.8	41.4	1
Mammogram	24.4	30.7	14	24.4	24.8	1
Bone Mineral Density	41.9	39.6	5	41.9	42.3	1

Data presented as percent except for age and Charlson Comorbidity Index which are presented as mean (standard deviation).

***Abbreviations***: Non-steroidal anti-inflammatory drug (NSAID)–excludes acetyl-salicylic acid, Acetylsalicylic acid (ASA), Histamine H2 Receptor antagonist (H2RA), Not applicable (N/A)

^a^ Weighted cohort based on inverse probability of treatment weights, using a propensity score based on 48 baseline characteristics.

^b^ Standardized differences are less sensitive to sample size than traditional hypothesis tests. They provide a measure of the difference between groups divided by the pooled standard deviation; a value greater than 10% is interpreted as a meaningful difference between the groups.

^c^ Defined as a population <10 000 people.

^d^ Income was categorized into fifths of average neighbourhood income on the cohort entry date.

^e^ The year of cohort entry is also referred to as the year of cohort entry date.

^f^ Comorbidities assessed by administrative database codes in the previous 5 years.

^g^ Charlson Comorbidity Index [Charlson ME, Pompei P, Alex KL, Mackenzie CR. A new method for classifying prognostic comorbidity in longitudinal studies: development and validation. J Chron Dis 1987;40(5):373–383. Quan H, Sundararajan V, Halfon P, Fong A, Burnand B, Luthi JC, et al. Coding algorithms for defining comorbidities in ICD-9-CM and ICD-10 administrative data. Med Care 2005;43(11):1130–1139.] was calculated using 5 years of hospitalization data. “No hospitalizations” received a score of 0.

^h^ The prevalence of depression is low since depression is not usually an in-patient disorder, and thus often not coded in the source databases.

^i^ Coronary artery disease includes receipt of coronary artery bypass graft surgery and percutaneous coronary intervention.

^j^ Major cancers include esophagus, lung, bowel, liver, pancreas, breast, male/female reproductive organs, as well as leukemias and lymphomas.

^k^ Baseline medication use assessed in the previous 120 days.

^l^ Excludes acetylsalicylic acid.

^m^ Refer to S5 Table for definitions of high and low doses.

^n^ Health care use assessed in the one year prior to SSRI prescription.

^o^ Based on the ICES physician database.

After weighting, with exception of the date of cohort entry, there was no significant difference between the two sets of comparison groups across all other 77 baseline characteristics measured in this study (see Tables 1 and 2). The year of cohort entry was expected to be different since escitalopram was added to Ontario’s provincial formulary in 2008. Only 6.5% of the citalopram group and 9% of the escitalopram group received prescriptions with high daily doses (defined in S5 Table; refer to S6 Table for yearly percentages).

### Outcomes

The primary and secondary outcomes are shown in Table 3 for citalopram and Table 4 for escitalopram. Across the entire cohort, in the 90-day follow up 140 patients (0.05%) had a record of a hospital encounter with ventricular arrhythmia and 8214 (3.01%) died.

**Table 3:** Relative risks for primary and secondary outcomes of patients prescribed citalopram compared to the referent antidepressants (paroxetine or sertraline).

	Number of events (%)			
Outcome	Citalopram n = 137 701	Paroxetine or Sertraline^b^ n = 135 746	Relative Risk (95% CI)	p-value
Ventricular Arrhythmia^a^	87 (0.06%)	56 (0.04%)	1.53 (1.03, 2.29)	0.04
All-Cause Mortality	4811 (3.49%)	4238 (3.12%)	1.12 (1.06, 1.18)	<0.01

Patients prescribed paroxetine or sertraline served as the comparator group.

**Abbreviations:** confidence interval (CI)

^a^ Based on hospital presentation (emergency room or hospitalization)–assessed by hospital diagnostic codes. This underestimated the true event rate because these codes tend to have high specificity but low sensitivity.

^b^ Weighted cohort and results based on inverse probability of treatment weights, based on a propensity score which used 48 baseline characteristics (see Methods section)

**Table 4:** Relative risks for primary and secondary outcomes of patients prescribed escitalopram compared to the referent antidepressants (paroxetine or sertraline).

	Number of events (%)			
Outcome	Escitalopram n = 38 436	Paroxetine or Sertraline^b^ n = 113 058	Relative Risk (95% CI)	p-value
Ventricular Arrhythmia^a^	13 (0.03%)	15 (0.04%)	0.84 (0.42, 1.68)	0.62
All-Cause Mortality	1100 (2.86%)	998 (2.63%)	1.09 (1.01, 1.18)	0.04

Patients prescribed paroxetine or sertraline served as the comparator group.

**Abbreviations:** confidence interval (CI)

^a^ Based on hospital presentation (emergency room or hospitalization)–assessed by hospital diagnostic codes. This underestimated the true event rate because these codes tend to have high specificity but low sensitivity.

^b^ Weighted cohort and results based on inverse probability of treatment weights, based on a propensity score which used 48 baseline characteristics (see Methods section)

The 90-day risk of ventricular arrhythmia in patients receiving citalopram was higher compared to referent antidepressants (0.06% vs. 0.04%; RR 1.53, 95% CI 1.03 to 2.29, p-value 0.04). Citalopram was also associated with a higher risk of all-cause mortality (3.49% vs. 3.12%, RR 1.12, 95% CI 1.06 to 1.18, p-value <0.01).

For escitalopram, the risk of ventricular arrhythmia compared to the referent antidepressants was not statistically different (0.03% vs. 0.04%; RR 0.84, 95% CI 0.42 to 1.68, p-value 0.62). Escitalopram was associated with a higher risk of all-cause mortality (2.86% vs. 2.63%; RR 1.09, 95% CI 1.01 to 1.18, p-value 0.04).

### Subgroup Analysis

**Fig 1:**
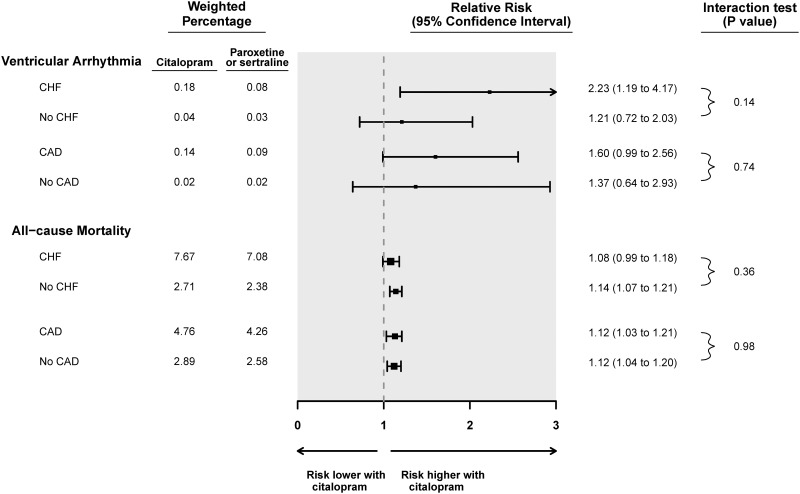
Subgroup analyses of the association between citalopram prescription and the risk of a hospital encounter with ventricular arrhythmia or all-cause mortality. **Abbreviations:** Coronary artery disease (CAD), Congestive heart failure (CHF), Confidence interval (CI).

**Fig 2:**
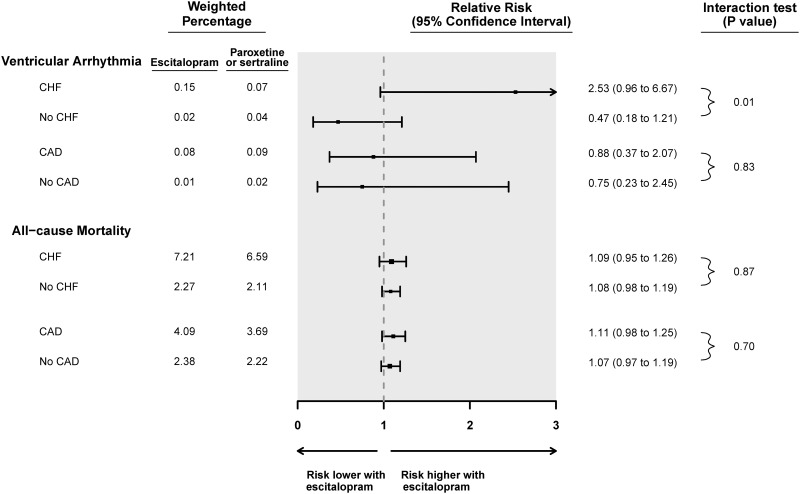
Subgroup analyses of the association between escitalopram prescription and the risk of a hospital encounter with ventricular arrhythmia or all-cause mortality. **Abbreviations:** Coronary artery disease (CAD), Congestive heart failure (CHF), Confidence interval (CI).

Subgroup analyses for ventricular arrhythmia and all-cause mortality are shown in Figs 1 and 2 for citalopram and escitalopram, respectively. The presence or absence of congestive heart failure did not significantly alter the association between citalopram and the risk of ventricular arrhythmia or all-cause mortality (p-values for interaction 0.14 and 0.36, respectively). Congestive heart failure did significantly modify the association between escitalopram compared to referent antidepressants and the risk of ventricular arrhythmia (p-value for interaction 0.01). Among those with congestive heart failure on escitalopram, the relative risk of ventricular arrhythmia was 2.53 (95% CI 0.96 to 6.67) whereas the relative risk was 0.47 (95% CI 0.18 to 1.21) for those without congestive heart failure. Congestive heart failure did not modify the association between escitalopram and all-cause mortality (p-value for interaction 0.87).

Coronary artery disease did not modify the association between SSRI drug and the risk of either ventricular arrhythmia or all-cause mortality (Figs 1 and 2). There were too few patients with chronic kidney disease or on high doses of SSRI to permit subgroup analysis.

## Discussion

In this population-based study of older adults newly prescribed SSRIs, we found that compared to paroxetine and sertraline, initiation of citalopram was associated with a small but statistically significant higher 90-day risk of a hospital encounter with ventricular arrhythmia. This increase in arrhythmia risk may have contributed to the observed small higher 90-day risk of death. Initiating citalopram compared to referent SSRIs was associated with a number needed to harm of 5000 (0.02% absolute increase) for the 90-day incidence of a hospital encounter with ventricular arrhythmia—assessed by hospital diagnostic codes. However, because hospital diagnostic codes are insensitive, the risk we observed is likely an underestimate of the true rate. Assuming the codes underestimate the incidence of arrhythmia by a factor of 10, the 90-day absolute risk increase would still be relatively low (1 in 500 patients). There were too few events in the escitalopram group to reliably assess the risk of ventricular arrhythmia. Thus, the higher association of 90-day all-cause mortality, and increased ventricular arrhythmia risk in the subgroup with congestive heart failure should be interpreted cautiously.

The findings of nine other studies (summarized in S7 Table) describing the association between citalopram or escitalopram and QT prolongation, ventricular arrhythmia, a cardiac event or mortality are inconsistent. We used the Downs and Black quality checklist to assess the reporting, external validity, internal validity and statistical power of these nine studies (S8 Table).[54] Based on this checklist, the quality was rated as good for two studies [5, 27], fair for five [14, 23, 26, 28–29] and poor for two (never published).[19,20] Out of the published studies, three focused on QT prolongation.[14, 26, 28] Compared to the other studies, our results on citalopram agree with the findings of Weeke et al (showing increased out-of-hospital cardiac arrest in a case-time-control study of elderly patients on citalopram),[29] but differ from those of two other cohort studies.[23, 27] Zivin *et al* showed no difference in the 5-year rates of ventricular arrhythmia and mortality in 618 450 US veterans who received a prescription for citalopram, compared to 365 898 patients with prescriptions for sertraline.[23] Leonard *et al* showed no difference in the 30-day rates of sudden death and ventricular arrhythmia in 294 434 United States Medicaid patients on citalopram compared to 560 822 patients on paroxetine.[27] Both these studies focused on a younger population (>50% were less than 65 years-old).

Escitalopram has previously been associated with a higher risk of the composite outcome of ventricular arrhythmia, cardiac arrest and sudden death in 14 128 pediatric patients, as compared to 32 906 pediatric patients prescribed fluoxetine.[5] We did not find a similar statistical association in our study, recognizing our observed rate of events was quite low.

Our study has several strengths: the use of Ontario’s healthcare databases allows for the assessment of all older residents who received the study antidepressants in routine care; our outcomes were clinically important adverse events; and we used a referent group who were also prescribed SSRIs and robust statistical methodology to balance the groups on 77 baseline characteristics, to reduce confounding.

Our study has limitations. Electrocardiograms were not available in our data sources; instead we relied on diagnostic codes for a hospital encounter with ventricular arrhythmia which have a good positive predictive value but limited sensitivity. However, we do not suspect any systematic difference in diagnostic recording by antidepressant type, suggesting that our relative measures of risk are robust. Also, our results may only generalize to older adults. As with any observational study residual confounding can never be fully eliminated.

Physicians who prescribe citalopram to older patients should be cognizant of the potential risk of ventricular arrhythmia and all-cause mortality. Our results suggest that in the elderly the warnings from regulatory agencies appear warranted. We detected a signal despite over 90% of our citalopram cohort taking a dose of ≤ 20mg/day (as per current recommendations). It is reassuring that the absolute increase in ventricular arrhythmia and mortality risk with citalopram was low. The FDA recommends monitoring of patients taking citalopram with electrocardiography;[19, 20] however, evidence for this approach is lacking.

In outpatient practice, we found a small increase in the 90-day risk of hospital encounter with ventricular arrhythmia in older adults prescribed citalopram compared to those prescribed paroxetine or sertraline. This may have contributed to the observed modestly higher risk of all-cause mortality with citalopram.

## Supporting Information

S1 Fig Cohort Selection. (DOC)

S1 Table Checklist of Recommendations for Reporting of Observational Studies Using the STROBE Guidelines. (DOCX)

S2 Table Database Codes for Baseline Characteristics. Abbreviations: CCI—Canadian Classification of Health Interventions (available after 2002), CCP—Canadian Classification of Diagnostic, Therapeutic and Surgical Procedures (before 2002), DSM—IV—Diagnostic and Statistical Manual of Mental Disorders IV coding, ICD 9 —International Classification of Diseases, Ninth Revision, ICD 10 —International Classification of Diseases, Tenth Revision. OHIP—Ontario Health Insurance Plan. * Where applicable a combination of diagnostic and procedure codes were used; ^a^ Treatment Code—from the Canadian Organ Replacement Register; ^b^ Treatment Organ—from the Canadian Organ Replacement Register: ^c^ Excluding cardiac angina: ^d^ DSM—IV—coding from Ontario Mental Health Reporting System: ^e^ Only includes dialysis visits with nephrologist present.
(DOCX)

S3 Table ICD 10 Codes for the Study Outcomes. ^a^ Only ICD 10 Codes were used to identify outcomes due to the timing of our study. These codes had to be associated with a hospital presentation in any position (e.g. most responsible diagnosis, or secondary diagnosis). ^b^ Data obtained from the Ontario Registered Persons Database and the Ontario Registrar General Death.
(DOCX)

S4 Table Variables included in the Propensity Score. Abbreviations: Selective serotonin re-uptake inhibitor (SSRI), Local Health Integration Network (LHIN). ^a^LHIN—Local Health Integration Network, health authorities responsible for regional administration of public healthcare services in Ontario; ^b^ computed tomography of a body area other than the head; ^c^ Refer to eTable 5 for the definitions of high and low doses.
(DOCX)

S5 Table High vs Low doses of Selective Serotonin Re-uptake Inhibitors. ** These doses are considered to be equivalent amongst the different types of selective serotonin re-uptake inhibitors.
(DOCX)

S6 Table Percentage of high dose citalopram (>20mg/day) or escitalopram (>10mg/day) prescriptions for each year prior to weighting. (DOCX)

S7 Table Summary of published studies describing ventricular arrhythmia/ QT prolongation/ Cardiac events/ mortality associated with citalopram or escitalopram use. Abbreviations: CI = confidence interval. ^a^We evaluated the quality of individual studies using the Downs and Black quality assessment method, which is a list of 27 criteria to evaluate both randomized and non-randomized trials (eTable 8) [57]. This scale assesses the completeness and clarity of study reporting, external validity, internal validity (e.g. bias and confounding) and power. The tool was modified slightly for use in our review. Specifically, the scoring for question 27 dealing with statistical power was simplified to a choice of awarding either 1 or 0 points depending on whether there was sufficient power to detect a clinically important effect. On the modified scale, we gave all included studies a score from 0 to 28, grouped into the following four quality levels: excellent (26 to 28), good (20 to 25), fair (15 to 19) and poor (less than 14).
(DOCX)

S8 Table Modified Downs and Black checklist for the assessment of the methodological quality of both randomized and non-randomized studies. ^a^Item has been modified; specifically, the scoring for this question was simplified to a choice of awarding either 1 or 0 points depending on whether there was sufficient power to detect a clinically important effect.
(DOCX)
