# Supplementary material for: Risk of Ventricular Arrhythmia with Citalopram and Escitalopram: A Population-Based Study
Source: PLoS One. 2016 Aug 11;11(8):e0160768. doi: 10.1371/journal.pone.0160768 (PMC4981428; doi:10.1371/journal.pone.0160768)
Supplement: S1 Fig — (DOC) [file pone.0160768.s001.doc]

Patients with evidence of a new outpatient oral prescription for a study SSRI between April 1^st^ 2002 and December 31st 2012.

N = 472 011

Patients excluded from study: (N = 199 254)

Missing age or sex: 9

Death on or before index date: 216

Non Ontario resident: 293

Evidence of antidepressants in the previous 180 days: 120 548

Non-standard daily dose of study antidepressant: 40 015

No previous Ontario Drug Benefit database prescriptions: 12 362

Hospital discharge in 2 days prior to their prescription date: 11 677

Evidence of ventricular arrhythmia in the past 5 years: 1 627

Evidence of cardiac arrest in the past 5 years: 481

Evidence of implantable defibrillator in the past 5 years: 593

Younger than 66 years old: 11 433

Patients included in final cohort

N = 272, 757

Citalopram 137 701

Escitalopram 38 436

Sertraline or paroxetine 96 620
