# Supplementary material for: Risk of Ventricular Arrhythmia with Citalopram and Escitalopram: A Population-Based Study
Source: PLoS One. 2016 Aug 11;11(8):e0160768. doi: 10.1371/journal.pone.0160768 (PMC4981428; doi:10.1371/journal.pone.0160768)
Supplement: S1 Table — (DOCX) [file pone.0160768.s002.docx]

|  | Item No | Recommendation | Reported |
| --- | --- | --- | --- |
| **Title and abstract** | 1 | (a) Indicate the study’s design with a commonly used term in the title or the abstract | Abstract |
|  |  | (b) Provide in the abstract an informative and balanced summary of what was done and what was found | Abstract |
| Introduction | | |  |
| Background/rationale | 2 | Explain the scientific background and rationale for the investigation being reported | Introduction |
| Objectives | 3 | State specific objectives, including any prespecified hypotheses | Introduction |
| Methods | | |  |
| Study design | 4 | Present key elements of study design early in the paper | Methods –Design and setting |
| Setting | 5 | Describe the setting, locations, and relevant dates, including periods of recruitment, exposure, follow-up, and data collection | Methods – Design and setting; Data sources |
| Participants | 6 | (a) Give the eligibility criteria, and the sources and methods of selection of participants. Describe methods of follow-up | Methods – participants |
|  |  | (b) For matched studies, give matching criteria and number of exposed and unexposed | Methods – Statistical analysis; Results |
| Variables | 7 | Clearly define all outcomes, exposures, predictors, potential confounders, and effect modifiers. Give diagnostic criteria, if applicable | Methods – Design and setting; Outcomes |
| Data sources/ measurement | 8 | For each variable of interest, give sources of data and details of methods of assessment (measurement). Describe comparability of assessment methods if there is more than one group | Methods – Data sources |
| Bias | 9 | Describe any efforts to address potential sources of bias | Methods – Statistical analysis; Discussion |
| Study size | 10 | Explain how the study size was arrived at | not applicable; use of existing health records |
| Quantitative variables | 11 | Explain how quantitative variables were handled in the analyses. If applicable, describe which groupings were chosen and why | not applicable |
| Statistical methods | 12 | (a) Describe all statistical methods, including those used to control for confounding | Methods – statistical analysis |
|  |  | (b) Describe any methods used to examine subgroups and interactions | Methods – participants; statistical analysis |
|  |  | (c) Explain how missing data were addressed | Not applicable |
|  |  | (d) If applicable, explain how loss to follow-up was addressed | Not applicable |
|  |  | (e) Describe any sensitivity analyses | Not applicable |
| Results | | |  |
| Participants | 13 | (a) Report numbers of individuals at each stage of study—e.g. numbers potentially eligible, examined for eligibility, confirmed eligible, included in the study, completing follow-up, and analysed | Results; eFig. 1 |
|  |  | (b) Give reasons for non-participation at each stage | eFig. 1 |
|  |  | (c) Consider use of a flow diagram | eFig. 1 |
| Descriptive data | 14 | (a) Give characteristics of study participants (e.g. demographic, clinical, social) and information on exposures and potential confounders | Methods – Participants; Results, Table 1 |
|  |  | (b) Indicate number of participants with missing data for each variable of interest | Methods – Data source (Complete with exception of specialty of prescribing physician and income quintile) |
|  |  | (c) Summarise follow-up time (e.g. average and total amount) | not applicable |
| Outcome data | 15 | Report numbers of outcome events or summary measures over time | Results; Table 2 and 3 |
| Main results | 16 | (a) Give unadjusted estimates and, if applicable, confounder-adjusted estimates and their precision (e.g. 95% confidence interval). Make clear which confounders were adjusted for and why they were included | Results; Table 2 and 3 |
|  |  | (b) Report category boundaries when continuous variables were categorized | not applicable |
|  |  | (c) If relevant, consider translating estimates of relative risk into absolute risk for a meaningful time period | Results; Table 2 and 3 |
| Other analyses | 17 | Report other analyses done—e.g. analyses of subgroups and interactions, and sensitivity analyses | Results; Fig. 1 and 2 |
| Discussion | | |  |
| Key results | 18 | Summarise key results with reference to study objectives | Discussion |
| Limitations | 19 | Discuss limitations of the study, taking into account sources of potential bias or imprecision. Discuss both direction and magnitude of any potential bias | Discussion |
| Interpretation | 20 | Give a cautious overall interpretation of results considering objectives, limitations, multiplicity of analyses, results from similar studies, and other relevant evidence | Discussion |
| Generalisability | 21 | Discuss the generalisability (external validity) of the study results | Discussion |
| Other information | | |  |
| Funding | 22 | Give the source of funding and the role of the funders for the present study and, if applicable, for the original study on which the present article is based | Cover page/Last Page |
