# Supplementary material for: Risk of Ventricular Arrhythmia with Citalopram and Escitalopram: A Population-Based Study
Source: PLoS One. 2016 Aug 11;11(8):e0160768. doi: 10.1371/journal.pone.0160768 (PMC4981428; doi:10.1371/journal.pone.0160768)
Supplement: S2 Table — Abbreviations: CCI—Canadian Classification of Health Interventions (available after 2002), CCP—Canadian Classification of Diagnostic, Therapeutic and Surgical Procedures (before 2002), DSM—IV—Diagnostic and Statistical Manual of Mental Disorders IV coding, ICD 9 —International Classification of Diseases, Ninth Revision, ICD 10 —International Classification of Diseases, Tenth Revision. OHIP—Ontario Health Insurance Plan. * Where applicable a combination of diagnostic and procedure codes were used; a Treatment Code—from the Canadian Organ Replacement Register; b Treatment Organ—from the Canadian Organ Replacement Register: c Excluding cardiac angina: d DSM—IV—coding from Ontario Mental Health Reporting System: e Only includes dialysis visits with nephrologist present. (DOCX) [file pone.0160768.s003.docx]

| **Baseline Characteristic** | **Codes^*^** |
| --- | --- |
| **Renal Disease** |  |
| Transplant |  |
| ICD 9 | V420, 99681 |
| ICD 10 | N165, Z940, T861 |
| OHIP Fee | E762, S435, E769, S434, E771, Z631, G347, G348, G412, G408, G409 |
| CCP | 6743, 675 |
| CCI | 1PC85 |
| Treatment Code^a^ | 171, 181 |
| Transplant Organ^b^ | 10, 11, 12, 18, 19 |
| Dialysis |  |
| OHIP Fee | G330, G331, G332, G861, G864, R849, G323, G325, G326, G860, G862, G863, G865, G866, G082, G083, G085, G090, G091, G092, G093, G094, G095, G096, G294, G295, G333 |
| CCP | 5195, 6698 |
| CCI | 1PZ21HPD4, 1KG76MZXXA, 1KG76MZXXN, 1PZ21HQBS, 1PZ21HQBR |
| Chronic Kidney Disease |  |
| ICD 9 | 4030, 4031, 4039, 4040, 4041, 4049, 582, 583, 580, 581, 584, 585, 586, 587, 5880, 5888, 5889, 5937 |
| ICD 10 | E102, E112, E132, E142, I12, I13, N08, N18, N19 |
| OHIP diagnosis | 403, 58 |
| Acute Kidney Injury |  |
| ICD 9 | 584 |
| ICD 10 | N17 |
| Hyperkalemia |  |
| ICD 9 | 2767 |
| ICD 10 | E875 |
| **Heart Diseases** |  |
| Congestive Heart Failure |  |
| ICD 9 | 425, 5184, 514, 428 |
| ICD 10 | I500, I501, I509, I255, J81 |
| OHIP diagnosis | 428 |
| OHIP Fee | R701, R702, Z429 |
| CCP | 4961, 4962, 4963, 4964 |
| CCI | 1HP53, 1HP55, 1HZ53GRFR, 1HZ53LAFR, 1HZ53SYFR |
| Coronary Artery Disease^c^ |  |
| ICD 9 | 412, 410, 414, 4292, 4295, 4296, 4297 |
| ICD 10 | I21, I22, I23, I24, I25, Z955, Z958, Z959, R931, T822 |
| OHIP diagnosis | 410, 412 |
| OHIP Fee | R741, R742, R743, G298, E646, E651, E652, E654, E655, G262, Z434, Z448 |
| CCP | 4801, 4802, 4803, 4804, 4805, 481, 482, 483 |
| CCI | 1IJ26, 1IJ27, 1IJ54, 1IJ57, 1IJ50, 1IJ76 |
| Acute Myocardial Infarction |  |
| ICD 9 | 410 |
| ICD 10 | I21, I22 |
| Angina |  |
| ICD 9 | 413 |
| ICD 10 | I20 |
| OHIP diagnosis | 413 |
| Pacemaker |  |
| OHIP Fee | G303, Z433, Z435, Z444, Z445, Z443, Z436, R752, Z412, Z428, E628, G176, G177, G115 |
| CCP | 4971, 4972, 4973, 0345, 0346, 0347, 0348, 0349, 4987 |
| CCI | 1HD53GRJA, 1HD54GRJA, 1HZ53GRNK, 1HZ53GRNL, 1HZ53GRNM, 1HZ54LANJ, 2HZ07NK, 2HZ07NL, 2HZ07NM, 1HZ53GRFR, 1HZ53LAFR, 1HZ53SYFR, 1HD55, 1HZ09, 1HZ55, 2HZ24, 1HZ37, 1HZ53GRNN, 1HZ53LANN |
| Atrial Fibrillation/Flutter |  |
| ICD 9 | 4273 |
| ICD 10 | I48 |
| Other Disorders |  |
| Dementia |  |
| ICD 9 | 2900, 2901, 2903, 2904, 2908, 2909, 2948, 2949, 3310, 3311, 3312, 2941, 797 |
| ICD 10 | F065, F066, F068, F069, F09, F00, F01, F02, F03, F051, G30, G31, R54 |
| OHIP diagnosis | 290,331, 797 |
| DSM – IV^d^ | 29040, 29041, 29042, 29043, 29120, 29282, 29410, 29411, 29480, 78090 |
| Schizophrenia or other psychotic disorders |  |
| ICD 9 | 2950, 2951, 2952, 2953, 2954, 2955, 2956, 2957, 2958, 2959, 2970, 2971, 2972, 2973, 2978, 2979, 2980, 2981, 2983, 2984, 2988, 2989 |
| ICD 10 | F060, F062, F105, F107, F115, F117, F125, F127, F135, F137, F145, F147, F155, F157, F165, F167, F175, F177, F185, F187, F195, F197, F200, F201, F202, F203, F204, F205, F206, F208, F209, F220, F228, F229, F230, F231, F232, F233, F238, F239, F24, F250, F251, F252, F258, F259, F28, F29 |
| OHIP diagnosis | 291, 292, 295, 297, 298 |
| OHIP Fee | Q021 |
| DSM – IV^d^ | 29130, 29150, 29211, 29212, 29381, 29382, 29510, 29520, 29530, 29540, 29560, 29570, 29590, 29710, 29730, 29880, 29890 |
| Bipolar Disorder |  |
| ICD 9 | 2960, 2961, 2964, 2965, 2966, 2967, 2968 |
| ICD 10 | F300, F301, F302, F308, F309, F310, F311, F312, F313, F314, F315, F316, F317, F318, F319 |
| OHIP diagnosis | 296 |
| OHIP Fee | Q020 |
| DSM – IV^d^ | 29600, 29601, 29602, 29603, 29604, 29605, 29606, 29640, 29641, 29642, 29643, 29644, 29645, 29646, 29650, 29651, 29652, 29653, 29654, 29655, 29656, 29660, 29661, 29662, 29663, 29664, 29665, 29666, 29670, 29680, 29689 |
| Unipolar depression or anxiety disorder |  |
| ICD 9 | 2962, 2963, 3000, 3002, 3003, 3004, 3091, 311 |
| ICD 10 | F063, F064, F320, F321, F322, F323, F328, F329, F330, F331, F332, F333, F334, F338, F339, F341, F400, F401, F402, F408, F409, F410, F411, F412, F413, F418, F419, F420, F421, F422, F428, F429, F430, F431 |
| OHIP diagnosis | 311 |
| DSM – IV^d^ | 29189, 29284, 29289, 29383, 29384, 29620, 29621, 29622, 29623, 29624, 29625, 29626, 29630, 29631, 29632, 29633, 29634, 29635, 29636, 30000, 30001, 30002, 30021, 30022, 30023, 30029, 30030, 30040, 30113 |
| Histoy of Self – Harm |  |
| ICD 9 | 9090, 9091, E950, E951, E952, E953, E954, E955, E956, E957, E958, E959 |
| ICD 10 | T961, T962, T969, T970, T971, T972, T979, X60, X61, X62, X63, X64, X65, X66, X67, X68, X69, X70, X71, X72, X73, X74, X75, X76, X77, X78, X79, X80, X81, X82, X83, X84, Y870, Z915 |
| Major Haemorrhage |  |
| ICD 9 | 430, 431, 432, 5307, 5310, 5312, 5314, 5316, 5320, 5322, 5324, 5326, 5330, 5332, 5334, 5336, 5340, 5342, 5344, 5346, 5780, 5781, 5693, 5789 |
| ICD 10 | I600, I601, I602, I603, I604, I605, I606, I607, I609, I61, 162, I850, I9820, I983, K2210, K2211, K2212, K2214, K2216, K226, K228, K250, K252, K254, K256, K260, K262, K264, K266, K270, K272, K274, K276, K280, K282, K284, K286, K290, K2921, K2941, K2951, K2961, K2971, K2981, K2991, K3180, K31811, K3182, K6380, K920, K921, K5520, K625, K922 |
| Haemorrhagic Stroke |  |
| ICD 9 | 430, 431 |
| ICD 10 | I600, I601, I602, I603, I604, I605, I606, I607, I609, I61 |
| Ischemic Stroke |  |
| ICD 9 | 436, 4340, 4341, 4349, 3623 |
| ICD 10 | I630, I631, I632, I633, I634, I635, I638, I639, I64, H341 |
| Transient Ischemic Attack |  |
| ICD 9 | 435 |
| ICD 10 | G450, G451, G452, G453, G458, G459, H340 |
| Chronic Liver Disease |  |
| ICD 9 | 4561, 4562, 070, 5722, 5723, 5724, 5728, 573, 7824, V026, 2750, 2751, 7891, 7895, 571 |
| ICD 10 | B16, B17, B18, B19, I85, R17, R18, R160, R162, B942, Z225, E831, E830, K70, K713, K714, K715, K717, K721, K729, K73, K74, K753, K754, K758, K759, K76, K77 |
| OHIP diagnosis | 571, 573, 070 |
| OHIP Fee | Z551, Z554 |
| Peripheral Vascular Disease |  |
| ICD 9 | 4402, 4408, 4409, 5571, 4439, 444 |
| ICD 10 | I700, I702, I708, I709, I731, I738, I739, K551 |
| OHIP Fee | R787, R780, R797, R804, R809, R875, R815, R936, R783, R784,R785, E626, R814, R786, R937, R860, R861, R855, R856, R933, R934, R791, E672, R794, R813, R867, E649 |
| CCP | 5125, 5129, 5014, 5016, 5018, 5028, 5038 |
| CCI | 1KA76, 1KA50, 1KE76, 1KG26, 1KG50, 1KG57, 1KG76MI, 1KG87 |
| Chronic Lung Disease |  |
| ICD 9 | 491, 492, 493, 494, 495, 496, 500, 501, 502, 503, 504, 505, 5064, 5069, 5081, 515, 516, 517, 5185, 5188, 5198, 5199, 4168, 4169 |
| ICD 10 | I272, I278, I279, J40, J41, J42, J43,J44, J45, J47, J60, J61, J62, J63, J64, J65, J66, J67, J68, J701, J703, J704, J708, J709, J82, J84, J92, J941, J949, J953, J961, J969, J984, J988, J989, J99 |
| OHIP diagnosis | 491, 492, 493, 494, 496, 501, 502, 515, 518, 519 |
| OHIP Fee | J889, J689 |
| Venous Thromboembolism |  |
| ICD 9 | 4511, 4512, 4151, 514 |
| ICD 10 | I26, I743, I801, I802, I803 |
| Cancer |  |
| ICD 9 | 150, 154, 155, 157, 162, 174, 175, 185, 203, 204, 205, 206, 207, 208 |
| ICD 10 | 971, 980, 982, 984, 985, 986, 987, 988, 989, 990, 991, 993, C15, C18, C19, C20, C22, C25, C34, C50, C56, C61, C82, C83, C85, C91, C92, C93, C94, C95, D00, D05 |
| OHIP diagnosis | 203, 204, 205, 206, 207, 208, 150, 154, 155, 157, 162, 174, 175, 183, 185 |
| Alcoholism |  |
| ICD 9 | 303, 3050 |
| ICD 10 | E24, E512, F10, G312, G621, G721, I426, K292, K70, K860, T510, X45, X65, Y15, Y573, Z502, Z714, Z721 |
| OHIP diagnosis | 303 |
| Seizure |  |
| ICD 9 | 345, 7803 |
| ICD 10 | G40, G41, R560, R568 |
| Consults |  |
| At Home Physician Services |  |
| OHIP Fee | A901, B960, B961, B962, B963, B964, B966, B990, B992, B993, B994, B996, B997, B998 |
| Nephrology Consult |  |
| OHIP Fee | C132, C101, C138, G860, G323, E083, C137, C135, A135, A161, A163, A164, A165, A166, A168 |
| Dialysis^e^ |  |
| OHIP Fee | R849, G323, G325, G326, G860, G862, G863, G865, G866, G330, G331, G332, G861,G864 |
| Procedures/ Interventions |  |
| Carotid Ultrasound |  |
| OHIP Fee | J201, J501, J189, J489', J190, J191, J490, J491, J492 |
| CCP | 0281 |
| CCI | 3JE30 |
| Cardiac Catheterization |  |
| OHIP Fee | G296, G297, G299, G300, G301, G304, G305, G306 |
| CCP | 4995, 4996, 4997 |
| CCI | 3IJ30GP, 3HZ30GP, 2HZ24GPKJ, 2HZ24GPKL, 2HZ24GPKM, 2HZ24GPXJ, 2HZ28GPPL, 2HZ71GP |
| Coronary Angiogram |  |
| OHIP Fee | G297, Z442 |
| CCP | 4892, 4893, 4894, 4895, 4896, 4897, 4898, 4996, 4997 |
| CCI | 3IP10 |
| Echocardiography |  |
| OHIP Fee | G560, G561, G562, G566, G567, G568, G570, G571, G572, G574, G575, G576, G577, G578, G579, G580, G581 |
| CCP | 0282 |
| CCI | 3IP30 |
| Holter Monitor |  |
| OHIP Fee | G650, G651, G652, G653, G654, G655, G656, G657, G658, GG59, G660, G661, G682, G683, G684, G685, G686, G687, G688, G689, G690, G692, G693 |
| CCI | 2HZ24JAKH |
| Cardiac Stress Test |  |
| OHIP Fee | G315, G174, G111, G112, G319, J604, J606, J607, J608, J611, J612, J613, J667, J807, J808, J809, J804, J811, J812, J813, J867, J609, J666, J866 |
| CCP | 0341, 0342, 0343, 0344 |
| CCI | 2HZ08, 3IP70 |
| Coronary Revascularization |  |
| OHIP Fee | R741, R742, R743, E651, E652, E654, E646, G298, Z434, G262 |
| CCP | 481, 482, 483, 480 |
| CCI | 1IJ50, 1IJ26, IIJ27, 1IJ57, 1IJ76 |
| Electrocardiography |  |
| OHIP Fee | G313, G310 |
| CCI | 2HZ24JAKE |
| Mammography |  |
| OHIP Fee | X172, X178, X184, X185, X201 |
| Bone Mineral Density |  |
| OHIP Fee | J654, J688, J854, J888, X149, X152, X153, X155, Y654, Y688, Y854, Y888 |
| Computed Tomography of Head |  |
| OHIP Fee | X188, X400, X401, X402, X405, X408 |
| Computed Tomography of other areas |  |
| OHIP Fee | X124, X125, X126, X127, X128, X231, X232, X233, X403, X404, X406, X407, X409, X410, X412, X413, X415, X416 |
| Chest X-ray |  |
| OHIP Fee | X090, X091, X092, X195 |
| Pulmonary Function Test |  |
| OHIP Fee | J301, J303, J304, J305, J306, J307, J308, J309, J310, J311, J313, J315, J316, J317, J318, J319, J320, J322, J323, J324, J327, J328, J330, J331, J332, J333, J334, J335, J340, J341, E450, E451 |
| Flu Shot |  |
| OHIP Fee | G590, G591 |
