# Supplementary material for: Risk of Ventricular Arrhythmia with Citalopram and Escitalopram: A Population-Based Study
Source: PLoS One. 2016 Aug 11;11(8):e0160768. doi: 10.1371/journal.pone.0160768 (PMC4981428; doi:10.1371/journal.pone.0160768)
Supplement: S3 Table — a Only ICD 10 Codes were used to identify outcomes due to the timing of our study. These codes had to be associated with a hospital presentation in any position (e.g. most responsible diagnosis, or secondary diagnosis). b Data obtained from the Ontario Registered Persons Database and the Ontario Registrar General Death. (DOCX) [file pone.0160768.s004.docx]

| **Outcome** | **ICD 10** |
| --- | --- |
| Ventricular Arrhythmia^a^ | I472, I4900 |
| All-Cause Mortality^b^ |  |
