# Supplementary material for: Risk of Ventricular Arrhythmia with Citalopram and Escitalopram: A Population-Based Study
Source: PLoS One. 2016 Aug 11;11(8):e0160768. doi: 10.1371/journal.pone.0160768 (PMC4981428; doi:10.1371/journal.pone.0160768)
Supplement: S4 Table — Abbreviations: Selective serotonin re-uptake inhibitor (SSRI), Local Health Integration Network (LHIN). aLHIN—Local Health Integration Network, health authorities responsible for regional administration of public healthcare services in Ontario; b computed tomography of a body area other than the head; c Refer to eTable 5 for the definitions of high and low doses. (DOCX) [file pone.0160768.s005.docx]

| **Variables included in the Propensity Score** | |
| --- | --- |
| Demographics | Age |
|  | Sex |
|  | Long term care facility |
|  | Rural region |
|  | LHIN^a^ |
| Comorbidities | Acute kidney injury |
|  | Angina |
|  | Cancer |
|  | Chronic lung disease |
|  | Coronary artery disease |
|  | Congestive heart failure |
|  | Dementia |
|  | Diabetes mellitus |
|  | Ischemic stroke |
|  | Hypertension |
|  | Major haemorrhage |
|  | Modified Charlson comorbidity score |
|  | Pacemaker |
|  | Chronic kidney disease |
| Medications | Acetyl-salicylic acid |
|  | Anticoagulants |
|  | Anti-arrhythmics |
|  | Anti-hypertensives |
|  | Anti-psychotics |
|  | Cholesterol lowering drugs |
|  | Non-steroidal anti-inflammatory drugs |
|  | Proton pump inhibitors |
|  | Histamine H2-receptor antagonists |
|  | Benzodiazepines |
| Investigations (a surrogate marker of health care access) | Computed Tomography of the head |
|  | Computed Tomography of other body area^b^ |
|  | Echocardiogram |
|  | Electrocardiogram |
|  | Holter monitor |
|  | Carotid ultrasound |
|  | Mammogram |
|  | Bone mineral density |
|  | Chest X-ray |
|  | Screening blood test |
| Interactions with Health Care | Cardiology visit |
|  | GP visit |
|  | Nephrology visit |
|  | Neurology visit |
|  | Psychiatry visit |
|  | Emergency room presentations |
|  | Hospitalizations |
| Other | Physician prescribing SSRI |
|  | High dose vs. low dose SSRI^c^ |
|  | Number of medications (polypharmacy) |
