# Supplementary material for: Risk of Ventricular Arrhythmia with Citalopram and Escitalopram: A Population-Based Study
Source: PLoS One. 2016 Aug 11;11(8):e0160768. doi: 10.1371/journal.pone.0160768 (PMC4981428; doi:10.1371/journal.pone.0160768)
Supplement: S5 Table — ** These doses are considered to be equivalent amongst the different types of selective serotonin re-uptake inhibitors. (DOCX) [file pone.0160768.s006.docx]

| **Drug** | **High dose (mg)**** | **Low dose (mg)**** |
| --- | --- | --- |
| Citalopram | >20 | ≤20 |
| Escitalopram | >10 | ≤10 |
| Sertraline | >50 | ≤50 |
| Paroxetine | >20 | ≤20 |
