# Supplementary material for: Risk of Ventricular Arrhythmia with Citalopram and Escitalopram: A Population-Based Study
Source: PLoS One. 2016 Aug 11;11(8):e0160768. doi: 10.1371/journal.pone.0160768 (PMC4981428; doi:10.1371/journal.pone.0160768)
Supplement: S6 Table — (DOCX) [file pone.0160768.s007.docx]

| **Year** | **Citalopram** | **Escitalopram** |
| --- | --- | --- |
| 2002 | 5.4% | N/A |
| 2003 | 5.3% | N/A |
| 2004 | 5.9% | N/A |
| 2005 | 5.9% | N/A |
| 2006 | 5.5% | N/A |
| 2007 | 5.6% | N/A |
| 2008 | 6.3% | 21.9% |
| 2009 | 7.9% | 9.0% |
| 2010 | 8.5% | 7.7% |
| 2011 | 8.9% | 8.4% |
| 2012 | 10.5% | 8.9% |
