# Supplementary material for: Risk of Ventricular Arrhythmia with Citalopram and Escitalopram: A Population-Based Study
Source: PLoS One. 2016 Aug 11;11(8):e0160768. doi: 10.1371/journal.pone.0160768 (PMC4981428; doi:10.1371/journal.pone.0160768)
Supplement: S7 Table — Abbreviations: CI = confidence interval. aWe evaluated the quality of individual studies using the Downs and Black quality assessment method, which is a list of 27 criteria to evaluate both randomized and non-randomized trials (eTable 8) [57]. This scale assesses the completeness and clarity of study reporting, external validity, internal validity (e.g. bias and confounding) and power. The tool was modified slightly for use in our review. Specifically, the scoring for question 27 dealing with statistical power was simplified to a choice of awarding either 1 or 0 points depending on whether there was sufficient power to detect a clinically important effect. On the modified scale, we gave all included studies a score from 0 to 28, grouped into the following four quality levels: excellent (26 to 28), good (20 to 25), fair (15 to 19) and poor (less than 14). (DOCX) [file pone.0160768.s008.docx]

| **Author** | **Study design/ Settings/ Participants** | **Exposures/ Timing/ Outcomes** | **Results for Citalopram/Escitalopram** | **Comments/ Limitations** | **Quality Score*** |
| --- | --- | --- | --- | --- | --- |
| ***Randomized Control Trials*** | | | | | |
| FDA (Citalopram Study)^19,20^ | - Randomized, double-blind, placebo-controlled, cross-over study  - 119 adult subjects | - Subjects received citalopram 20mg and 60mg, moxifloxacin 400mg and placebo (per day)  - Effect of citalopram on QTc prolongation at different doses | - At 20mg/day citalopram increased the QTc by 8.5ms (90% CI, 6.2 to 10.8)  - At 60mg/day citalopram increased the QTc by 18.5ms (90% CI, 16.0 to 21.0)  - Moxifloxacin at 400mg/day increased the QTc by 13.4ms (90% CI, 10.9 to 15.9) | - Surrogate outcome of QTc prolongation was used, as opposed to clinically relevant outcomes such as arrhythmia  - Study never published – as such, details about the methodology, subjects’ baseline characteristics, and analyses are missing. | 6 |
| FDA (Escitalopram Study)^20^ | - Randomized, double-blind, placebo-controlled, cross-over study  - 113 adult subjects | - Subjects received escitalopram 10mg and 30mg, moxifloxacin 400mg and placebo (per day)  - Effect of escitalopram on QTc prolongation at different doses | - At 10mg/day escitalopram increased the QTc by 4.5ms (90% CI, 2.5 to 6.4)  - At 30mg/day escitalopram increased the QTc by 10.7ms (90% CI, 8.7 to 12.7)  - Moxifloxacin at 400mg/day increased the QTc by 9.0ms (90% CI, 7.3 to 10.8) | - Surrogate outcome of QTc prolongation was used, as opposed to clinically relevant outcomes such as arrhythmia  - Study never published – as such, details about the methodology, subjects’ baseline characteristics, and analyses are missing. | 6 |
| ***Population based studies*** | | | | | |
| Castro 2013^14^ | -Retrospective cross sectional study  - New England healthcare system: 2 academic centers and outpatient clinics  -38 397 adult patients with an electrogram recorded after prescription of antidepressant or methadone between Feb 1990 – Aug 2011 | - Patients were initiated on antidepressants or methadone therapy  - Dose-response association between QTc interval prolongation 14-90 days after a prescription (and at maximum dose) | - Dose-response association with QTc prolongation was identified with citalopram (adjusted beta 0.1, p<0.01), escitalopram (adjusted beta 0.58, p<0.001), and amitriptyline (adjusted beta 0.11, p<0.001).  - Within subject paired observations supported QTc prolonging effect of citalopram (10-20mg, mean QTc increase 7.8ms, adjusted p<0.05; and 20-40mg, mean QTc increase 10.3ms, adjusted p<0.01). | - Linear regression models included dose of medication, age, sex, health insurance, ethnicity, cardiovascular disease history, use of methadone or antipsychotic, and diagnosis of major depressive disorder  - No controls  - Surrogate outcome of QTc prolongation is an imperfect predictor of ventricular arrhythmia  - Source bias: patients receiving electrocardiograms might be at higher risk | 16 |
| Czaja 2013^5^ | - Retrospective cohort study  - US health insurance claims data from 1997 to 2009  - 113,714 new pediatric (age <18years) users of SSRI monotherapy; 14,128 used citalopram, 14,079 escitalopram and 32,906 fluoxetine. | - Exposures: new prescription for SSRI monotherapy compared to referent SSRI fluoxetine  - Outcomes: composite of ventricular arrhythmia, cardiac arrest and sudden death occurring within 12 months of SSRI initiation | - The adjusted risk of adverse event for citalopram showed a hazard ratio 3.53 (95% CI 1.09 to 11.46) compared to fluoxetine  - The adjusted risk of adverse event for escitalopram showed a hazard ratio 3.30 (95% CI 1.08 to 10.14) compared to fluoxetine | - Adjusted for propensity to receive an individual SSRI, demographics, and exposure covariates  - Health insurance claims data miss patients from lower socio-economic background who may not have insurance  - Focus on pediatric population | 20 |
| Leonard 2011^27^ | - Retrospective cohort study  - Medicaid and Medicare claims data from beneficiaries of California, Florida, Ohio, New York and Pennsylvania, USA  - Cohort consisted of 1,287,446 person-years of exposure to 21 different antidepressants; Citalopram was used by 294 434 patients, and paroxetine by 560 822 patients. | - Exposure to study antidepressants (included citalopram and sertraline) compared to the control antidepressant, paroxetine.  - Outcomes: incident emergency department or inpatient diagnosis of sudden death or ventricular arrhythmia originating in the outpatient settings in patients prescribed 20 different antidepressants compared to paroxetine | - Adjusted hazard ratio for sudden death or ventricular arrhythmia for citalopram compared to paroxetine was near unity (1.0) | - Hazard ratio only adjusted for age, sex, race, state, nursing home residence, bipolar disorder diagnosis, angiotensin-converting enzyme inhibitor or angiotensin-II-receptor blocker prescription. (Other covariates affected the hazard ratio <10%, and thus were excluded.)  - Users were predominantly females and non-elderly | 20 |
| Uchida 2015^28^ | - Retrospective cross sectional study  - Electronic medical records in the Partners Healthcare system, Massachusetts, USA  - 297 children aged 5-18 years old with at least one prescription for an antidepressant or methadone between 1990 and 2011 | - Exposure: anti-depressants (including citalopram, escitalopram, paroxetine, sertraline, fluoxetine, bupropion, duloxetine, venlafaxine, mirtazapine, amitriptyline, and nortriptylene) and methadone  - Outcome: QTc prolongation on electrocardiograms received between 14-90 days after prescription | - The highest mean QTc was in patients on escitalopram (436ms). Mean QTc values for all medications were in the normal range.  - The differences in QTc did not reach statistical significance, except for sertraline which had a mean QTc 10.6ms shorter than all other study drugs (p<0.05).  - There were no documented ventricular arrhythmias | - Used surrogate outcomes, not clinically relevant ones  - Adjusted QTc was generated using linear models accounting for potential confounding effects (age at prescription, gender, race, insurance, year of prescription, history of myocardial infarction, hypertension, hyperlipidemia, ventricular arrhythmia and major depressive disorder).  - No control group  - Limited power at 62% - since few children get electrocardiograms  - Pediatric population | 14 |
| Van Haelst 2013^26^ | - Retrospective cross sectional study  - University Medical Center Utrecht, Netherlands – hospital databases  - Subjects: >60 years old individuals scheduled for outpatient pre-anesthesia evaluation between 2007 and 2012  - Index group included 397 users of SSRIs, and the reference group 397 non-users, matched on sex and year of scheduled surgery  - Reference group was randomly selected to match the index group on a 1:1 ratio | - Index group were on SSRIs (including citalopram, escitalopram, paroxetine and sertraline), while the reference group was not on anti-depressants  - Primary outcome was QTc prolongation. Secondary outcome was duration of the QTc interval. | - Users of SSRI did not have a higher risk for QTc prolongation (OR 1.1, 95% CI 0.5 to 2.0)  - Use of citalopram or paroxetine was not associated with prolonged QTc | - Confounders assessed: weight, height, smoking, alcohol abuse, pre-operative physical status, atrial fibrillation, heart failure, diabetes, hypertension, myocardial infarction, pulmonary disease, and co-medications  - Outcome was QTc prolongation, an imperfect surrogate for ventricular arrhythmia  - Low power | 18 |
| Weeke 2012^29^ | - Retrospective case-time-control study  - Danish Cardiac Arrest Register and the Copenhagen Emergency Care unit, Denmark  - 19,110 patients (older than 9 years on Jan 1^st^ 1997), who had an out-of-hospital cardiac arrest between 2001 and 2007, out-of-which 2,913 patients were on any antidepressant at the time of the event | - Study group consisted of patients who had an out-of-hospital cardiac arrest and who were on anti-depressants at the time of the arrest.  - The case period was 30-0 days before the event, while the control periods were 120-90 and 90-60 days before the event (for case-time-control analysis)  - Control group identified from the entire Danish population and matched (1:4) on age and sex  - Outcome: association between out-of-hospital cardiac arrest and use of antidepressant | - Median age 70.5 years  - Patients having an out-of-hospital cardiac arrest had an odds ratio of 1.29 (95% CI: 1.02 to 1.63) of being on citalopram, and an odds ratio of 1.10 (95% CI 0.64-1.87) of being on escitalopram (using case-time-control models). | - Compared the medications received at specific time periods before cardiac arrest using conditional logistic regression analysis, providing good control for chronic conditions, but not for the risk associated with the indication for the medication  - The control group was only matched to the study group by age and sex, thus there may have been significant differences between their baseline characteristics  - Despite two different comparison groups (control group and case-time-control analysis), the study did not account for confounding by indication. Depression in itself has been associated with sudden cardiac death and increased mortality.  - Accuracy of out-of-hospital events recording is unclear | 15 |
| Zivin 2013^23^ | - Retrospective cohort study  - Veterans Health Administration National Registry for Depression data between 2004 and 2009  - 618,450 depressed adult patients receiving a prescription for citalopram and 365,898 receiving a prescription for sertraline | - Patients received a prescription for citalopram or sertraline at different doses  - Observation time: from first prescription to time of event or end of study (2009)  - Outcomes: association of antidepressant dosing with ventricular arrhythmia, and cardiac, non-cardiac and all-cause mortality | - Citalopram daily doses >40 mg were associated with lower risks of ventricular arrhythmia (adjusted hazard ratio 0.68, 95% CI 0.61–0.76), all-cause mortality (adjusted hazard ratio 0.94, 95% CI 0.90–0.99), and non-cardiac mortality (adjusted hazard ratio 0.90, 95% CI 0.86–0.96) compared with daily doses of 1–20 mg.  - Citalopram daily doses of 21–40 mg were associated with lower risks of ventricular arrhythmia (adjusted hazard ratio 0.80, 95% CI 0.74–0.86) compared with dosages of 1–20 mg/day but did not have significantly different risks of any cause of mortality.  - No increased risks of cardiac mortality were found.  - The sertraline cohort had similar lower risk of ventricular arrhythmia at higher doses, and no significant association between daily dose and either all-cause or non-cardiac mortality | - Patient population consisted of mostly white (72.5%), non-Hispanic (84%), males (90.4%), with a mean age of 56.9 years.  - Adjusted Cox models only controlled for sociodemographic data and comorbidity score (not specific risk factors)  - Only looked at patients diagnosed with depression.  - Cardiac outcomes may be misclassified and administrative data underreported. | 16 |
